# Publisher’s Note

**DOI:** 10.1080/21655979.2024.2326357

**Published:** 2024-03-01

Due to a publisher error, a retraction notice was incorrectly issued on the article ‘The mechanism of α2-macroglobulin against oxidative stress and promoting cell proliferation in intervertebral disc degeneration’, https://doi.org/10.1080/21655979.2021.2011638. We have now removed the retraction notice.

We apologise to the authors for this error.

